# Over-Expression of PUMA Correlates with the Apoptosis of Spinal Cord Cells in Rat Neuropathic Intermittent Claudication Model

**DOI:** 10.1371/journal.pone.0056580

**Published:** 2013-05-02

**Authors:** Bin Ma, Jiangang Shi, Lianshun Jia, Wen Yuan, Jianfeng Wu, Zhiyi Fu, Yuan Wang, Ning Liu, Zhengmao Guan

**Affiliations:** 1 Department of Spine Surgery, Bone and Joint Medical Center, East Hospital, Tongji University School of Medicine, Shanghai, China; 2 Division of Orthopedics, Orthopedics Institute of PLA, Changzheng Hospital, Second Military Medical University, Shanghai, China; Hertie Institute for Clinical Brain Research and German Center for Neurodegenerative Diseases, Germany

## Abstract

**Background:**

Neuropathic intermittent claudication (NIC) is a typical clinical symptom of lumbar spinal stenosis and the apoptosis of neurons caused by cauda equina compression (CEC) has been proposed as an important reason. Whereas, the factors and the mechanism involved in the process of apoptosis induced by CEC remain unclear.

**Methodology and Results:**

In our modified rat model of NIC, a trapezoid-shaped silicon rubber was inserted into the epidural space under the L5 and L6 vertebral plate. Obvious apoptosis was observed in spinal cord cells after compression by TUNEL assay. Simultaneously, qRT-PCR and immunohistochemistry showed that the expression levels of PUMA (p53 up-regulated modulator of apoptosis) and p53 were upregulated significantly in spinal cord under compression, while the expression of p53 inhibitor MDM2 and SirT2 decreased in the same region. Furthermore, CEC also resulted in the upregulation of Bcl-2 pro-apoptotic genes expression and caspase-3 activation. With the protection of Methylprednisolone, the upregulation of PUMA and p53 expression as well as the decrease of MDM2 and SirT2 in spinal cord were partially rescued in western bolt analysis.

**Conclusions:**

These results suggest that over-expression of PUMA correlates with CEC caused apoptosis of spinal cord cells, which is characterized by the increase of p53, Bax and Bad expression. PUMA upregulation might be crucial to induce apoptosis of spinal cord cells through p53-dependent pathway in CEC.

## Introduction

Neurogenic intermittent claudication (NIC) is the most common presenting symptom in lumbar spinal canal stenosis, which is caused by compression of the cauda equina. NIC is characterized by the occurrence of intermittent pain in lower extremities or in the lower back and is typically exacerbated with prolonged walking or lumbar extension [1], [2]. In view of the poor understanding of the pathogenesis and molecular mechanism of this disease, the therapy strategy for NIC is very limited.

Several rat models mimicking NIC have been established to study the pathophysiology of the polyradicular symptomatology for NIC patients [3], [4]. These studies reveal that a reduction in blood flow in compressed nerve roots and the development of local spinal ischemia might represent partial mechanisms for the clinical symptom of NIC [3], [5], [6]. Recently, apoptosis of motor neurons has been suggested to be an important reason for dysfunctions of NIC [4], [7]. However, it is rarely known about the factors involved in this apoptotic process. PUMA (p53 up-regulated modulator of apoptosis) has been suggested to be critical for caspase-3 activation and neuronal apoptosis [8], [9]. Whether PUMA is essential for compression induced apoptosis is valuable to be explored.

In this study, we report a modified rat model of NIC with easier manipulation and better healthy, named as modified cauda equina compression model (MCC). Further study showed that the apoptosis in spinal cord cells were highly associated with the overexpression of PUMA with region specificity. Additionally, p53 and Bcl-2 pro-apoptotic genes were upregulated in the same region, accompanying with the decrease of p53 inhibitors SirT2 and MDM2. It demonstrates that compression elicited apoptosis in spinal cord undergoes p53-PUMA dependent pathway to activate caspase-3 activity and sequential apoptotic events.

## Materials and Methods

### Animals

A total of 168 adult male Sprague-Dawley rats (8–9 week old, 200–250 g) were used in our study. All animal experiments conformed to the regulations of the Animal Research Committee of Second Military Medical University and accorded with the Guidelines on Animal Experiments at Second Military Medical University and the Chinese Government Animal Protection and Management Law.

### Surgical procedures

The rats were randomly divided into three groups: sham-operated group (SHAM), n = 8; classic cauda equina compression group (CCC), n = 8; modified cauda equina compression group (MCC), n = 8. Rats were anesthetized with 3% sodium pentobarbital, 35 mg/kg by intraperitoneal injection. Then, the rat was placed into a prone position and incised along the midline of spine. L4, L5 and L6 vertebral bodies were exposed using microscopy and drilled through L4/5 and L5/6. In CCC model, two silicon rubbers were inserted (4.0×1.0×1.25 mm) to the upper and lower caudal end, respectively [4]. After surgery, the incision was sutured and scattered with penicillin to prevent infection.

**Figure 1:**
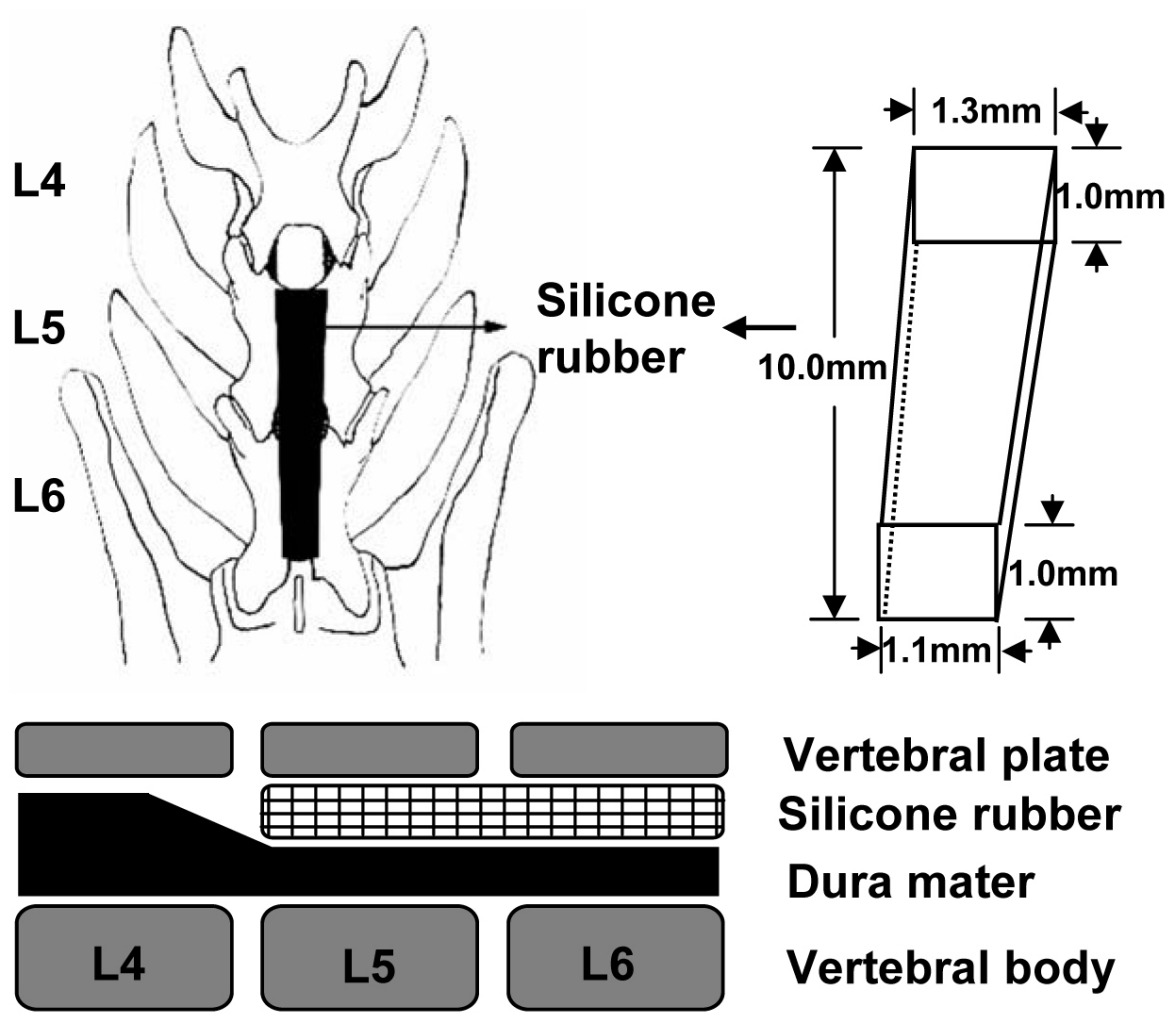
Surgical procedure of modified cauda equina compression. After exposure of L4 and L5 vertebral plates, a piece of trapezoid-shaped silicon rubber (10.0×1.0×1.1 to 10.0×1.0×1.3 mm) was inserted into the epidural space under the L5 and L6 vertebral plate.

In our modified cauda equina compression model, the rats were revealed L4 and L5 vertebral plates. The ligamentum flavum between L4 and L5 was removed. Subsequently a piece of trapezoid-shaped silicon rubber (10.0×1.0×1.1 to 10.0×1.0×1.3 mm) was inserted into the epidural space under the L5 and L6 vertebral plate. From preliminary analysis, the silicone rubber was known to occupy about 36% of the spinal canal area at L5 and L6 (Fig. 1). Be sure not to injure the dural sac during the operation. In sham-operated group, rats were only posterior open and drilled, without inserting silicon rubbers and forming compression. It was used to exclude the effects of anesthesia and operation.

### Behavioral Assessment of the Animal Model

After surgery, sensory functions of each group were tested by measuring thermal and tactile withdrawal threshold following the methods described by Takenobu et al [4], Basso, Beattie, and Bresnahan (BBB) score [10] and walking distance for the period from 3 days before operation to 28 days after operation, which was day −3, 1, 3, 7, 14, and 28. Walking distance was measured using a treadmill apparatus. Forced running distance and free running distance were measured according to the protocol described in previous reports [4]. All rats underwent daily exercise initiated 3 days before surgery. At the day 1, 3, 7, 14 and 28 after surgery, the walking distance was measured.

### TUNEL Assay

The apoptosis of cellula nervosa were detected by TdT-mediated X-dUTP nick end labeling (TUNEL) assay described as follows: the sections were placed onto glass-slides. Following deparaffinization in xylene, the slides were rehydrated and washed with Tris Buffered Saline (TBS). The endogenous peroxidase activity was quenched by 5 min incubation in a mixture of 3% hydrogen peroxide solution with protease K. Then the slides were kept in TUNEL mixed liquor for one hour and Streptavidin-HRP for 30 min. After washing with TBS, the slides were kept in diaminobenzidine for 5–10 min and counterstained with Mayers hematoxylin. The slides were sealed with resin after blow-drying, and then observed and photographed with the optical microscope. Apoptosis-positive cells were counted in three sections per rat. Numbers of apoptosis-positive cells in >5 fields of vision under high power lens were calculated and the average number per field were shown.

### Quantitive Realtime PCR (qRT-PCR)

The mRNA expression levels in spinal cord (SC), cauda equina (CE) and dorsal root ganglion (DRG) were detected using qRT-PCR. Total RNA was extracted from the tissues using Trizol reagent (Invitrogen, USA). The content of total RNA was calculated with ultraviolet spectrophotometer. Reverse transcription was performed using M-MLV reverse transcriptase (Promega, USA), and the cDNA product was then quantified by qRT-PCR with the following primers:

PUMA forward: ATCTCTTCATGGGACTCCTC, reverse: GGCAGTCCAGTATGCTACATG;

β-actin forward: TGTTGTCCCTGTATGCCTCTGGTC; reverse: ATGTCACGCACGATTTCCCTCTCA;

Bcl-2 forward: GCTACGAGTGGGATACTGG; reverse: GTGTGCAGATGCCGGTTCA;

Bcl-xl forward: AGGATACAGCTGGAGTCAG; reverse: TCTCCTTGTCTACGCTTTCC;

Bax forward: CTGCAGAGGATGATTGCTGA; reverse: GATCAGCTCGGGCACTTTAG;

Bad forward: TCCAGCTAGGATGATAGGAC; reverse: TCCAGCTAGGATGATAGGAC;

Bak forward: CAGCTTGCTCTCATCGGAGAT; reverse: GGTGAAGAGTTCGTAGGCATTC;

The LightCycler software 3.0 automatically evaluated the data acquired and the cycle threshold (Ct) values were obtained and normalized to β-actin by the 2^−ΔΔCt^ method.

### Immunohistochemistry

The expression levels of PUMA (Cell Signaling, 1∶200), p53 (Santa Cruz Biotechnology, 1∶300), and SirT2 (Santa Cruz Biotechnology, 1∶50) in SC, CE and DRG were detected by immunohistochemical methods described as follows: the sections were placed onto glass-slides. Following deparaffinization in xylene, the slides were rehydrated and washed with TBS. The endogenous peroxidase activity was quenched by incubation in a mixture of 3% hydrogen peroxide solution for 5 min. After being boiled in citrate buffer pH 6.0 for 20 min, sections were sealed and kept in 10% nonimmune goat serum in TBS (pH 7.5) at room temperature for 20 min. After that, they were incubated with primary antibody at room temperature for 1 h, then with secondary antibody at room temperature for 30 min. After washing with TBS, the slides were kept in diaminobenzidine for 5–10 min and counterstained with Mayers hematoxylin. All the specimens were observed and photographed by microscope.

### Western Blot

Spinal cord tissues with dorsal and ventral horn neurons (100 mg) were homogenized in Lysis Buffer containing 50 mM Tris•Cl (pH7.5), 150 mM NaCl, 1% NP-40, 0.5% sodium desoxycholate (w/v), 0.1% SDS (w/v), 10 mM mercaptoethanol, 10 mg/ml PMSF, 5 µg/ml Pepstatin, and 5 µg/ml leupeptin (pH7.4). Electrophoresis of homogenated aliquots was carried out using polyacrylamid gels. Separated proteins were then electrotransferred to the nitrocellulose membranes. These membranes were incubated in primary antibody for 24 h at 4°C and subsequently in the secondary peroxidase-conjugated antibody for 2 h. Immunoreactive protein bands were visualized using an enhanced chemiluminescence reaction kit (PIERCE, USA). Meanwhile β-actin was used as loading control. The following primary antibodies were used: PUMA (Cell Signaling, 1∶1000), p53 (Santa Cruz Biotechnology, 1∶2000), pro-caspase-3 (Abcam, 1∶500), active caspase-3 (Cell Signaling, 1∶500), MDM2 (Santa Cruz Biotechnology, 1∶800), SirT2 (Santa Cruz Biotechnology, 1∶500), Tuj1 (Santa Cruz Biotechnology, 1∶1000) and β-actin (Sigma, 1∶10000).

### Statistical analysis

The software SPSS 11.0 was applied for the statistical analysis. All data were reported as mean ±SD. Differences among groups were analyzed using One-Way ANOVA method. If the homogeneity of variance and the normality assumption were not verified, a non parametric test was used. All tests were two-sided with a significance level of p value<0.05 (*).

## Results

### Behavioral Assessment of the Animal Model

All experimental groups, behavioral assessments including motor and sensory testing were performed before surgery and in specific time points after surgery between 1 and 28 days. All experimental rats showed low death rate and a few of exceptions such as rats with huge hematoma were excluded in behavioral test. The weight gains of the rats were normal, and no statistical difference was found between each group (p>0.05) (data not shown).

**Figure 2:**
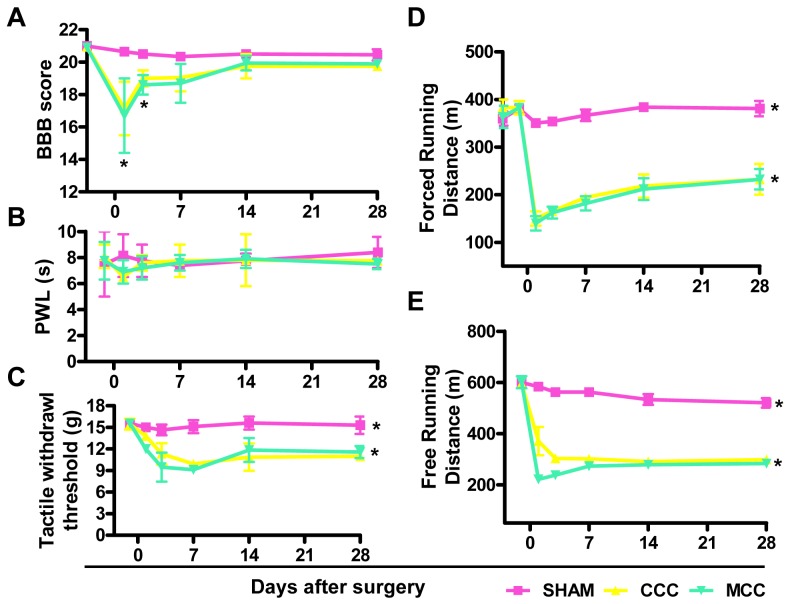
Behavioral Assessment of the Animal Model. (A–C) BBB scores (A), paw withdrawal threshold (PWL) under thermal stimulation (B) and tactile withdrawal threshold (C) were determined at different time points before or after surgery (day −3/−1, 1, 3, 7, 14, 28) in sham-operated group (SHAM), classic cauda equina compression group (CCC) and modified cauda equina compression group (MCC). (D–E) Forced running (D) or free running (E) distances were tested in SHAM, CCC and MCC group rats.

BBB scores of each group were all 21 before surgery. The rats of Sham-operated group gradually recovered their motor function of hind limbs, and achieved to preoperative level in one day. Both CCC and MCC groups had tail flick dysfunction one day after surgery, and recovered to different degrees in 28 days. Additionally, there was a statistical difference between MCC/CCC and SHAM groups in BBB score (p<0.05) at day 1 and 3 (Figure 2A), suggesting that important events might happen around day 3.

Paw-withdrawal latency was measured under thermal stimulation and no significant differences in the thermal withdrawal threshold were observed between experimental groups (Figure 2B), which is similar with previous reports [4]. We also examined tactile withdrawal threshold and no detectable changes were found in SHAM group, while a significant tactile allodynia was generated in both MCC and CCC animals through 28 days (p<0.05) (Figure 2C). No statistical difference was detected between CCC and MCC groups (p>0.05) (Fig. 2).

Walking distances of all groups displayed no statistical difference before surgery and walking ability of SHAM group was not affected by surgery. Walking dysfunction was observed in group CCC and MCC after surgery when compared with that in sham-operated rats (p<0.05) in either free or forced running distance test, and there was no significantly difference between CCC and MCC groups on any measured time (p>0.05) (Figure 2D, E).

### Compression caused apoptosis in spinal cord cells

**Figure 3:**
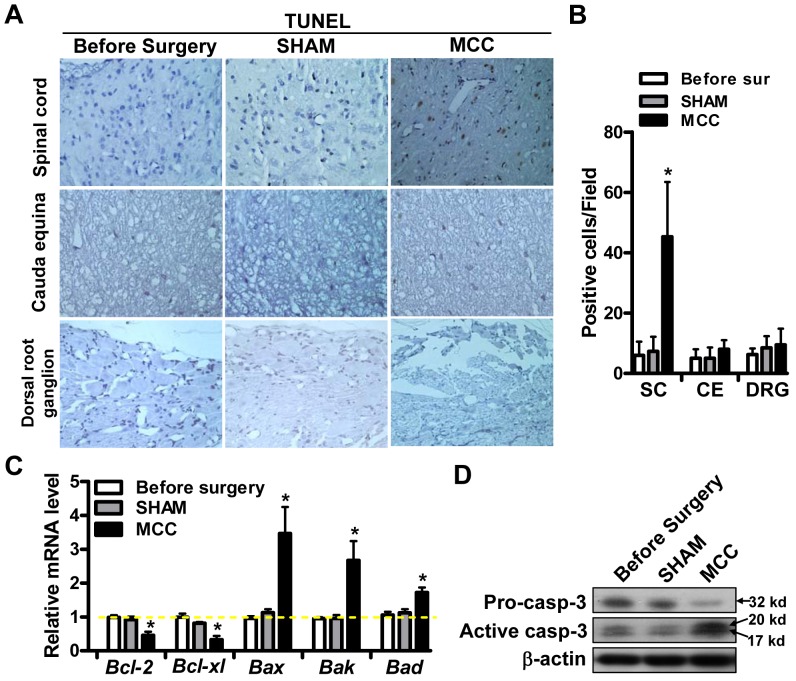
Compression caused apoptosis in spinal cord cells. (A) The apoptosis of cells in spinal cord (SC), cauda equina (CE) and dorsal root ganglion (DRG) were analyzed by TUNEL method in three groups: before surgery; sham-operated; MCC group. (B) TUNEL positive cells were counted in >5 fields and the average numbers were shown in different groups. (C) qRT-PCR analysis of the expression of Bcl-2, Bcl-xl, Bax, Bak and Bad in three experimental groups. (D) The expression level of pro-caspase-3 (pro-casp-3) and active caspase-3 (active caspase-3) was determined by western blot in indicated 3 groups.

It has been proposed that neuronal apoptosis is an important reason for dysfunctions of NIC [7], which drives us to check the cell apoptosis in different regions of the brain by TUNEL assay. Obviously more apoptosis-positive cells at day 3 after surgery were detected in spinal cord (SC) after surgery when compared with that before surgery (Figure 3A), which was confirmed by statistical analysis (p<0.05) (Figure 3B). Whereas, apoptotic cells were rarely detected in cauda equina (CE) and dorsal root ganglion (DRG) in all experimental groups (Figure 3A, B).

Furthermore, the expression of apoptosis related genes was determined in spinal cord. Among the Bcl-2 family members, the expression of pro-survival genes Bcl-2 and Bcl-xl was downregulated in MCC comparing with that before surgery or in SHAM group (Figure 3C). Conversely, the pro-apoptotic genes (Bax, Bak and Bad) expression was increased after compression (Figure 3C). Notably, western blot analysis showed that the expression level of cleaved active caspase-3 was significantly increased (Figure 3D). Based on these observations, we populate that compression caused apoptosis in spinal cord cells might undergo classical caspase dependent pathway.

### Increased expression of PUMA correlates with apoptosis of spinal cord cells

**Figure 4:**
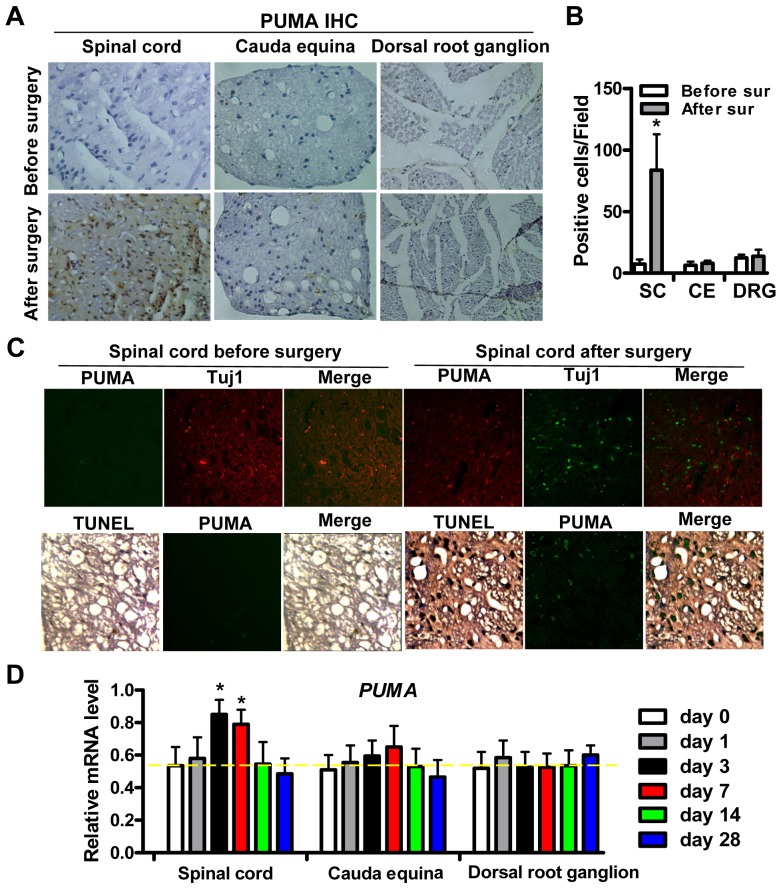
Increased expression of PUMA correlates with apoptosis of spinal cord cells. (A) The expression of PUMA in spinal cord (SC), cauda equina (CE) and dorsal root ganglion (DRG) was examined using immunohistochemistry (IHC) before (day -3) or after surgery (day 3). (B) 5 independent fields of PUMA IHC staining were counted and the average numbers of PUMA-positive cells were shown. (C) Double staining of Tuj1 (Red) and PUMA (Green) in spinal cord before or after surgery. In the lower panel, TUNEL assay and sequential PUMA staining (Green) were performed in the same specimens from spinal cord region before or after surgery. (D) qRT-PCR analysis of PUMA mRNA level at different time points before (day 0) or after surgery (day 1, 3, 7, 14, 28) in spinal dorsal, cauda equina, and dorsal root ganglion.

PUMA has been suggested to be critical for caspase-3 activation and neuronal apoptosis [8], [9], the expression of PUMA was determined by immunohistochemistry (IHC). The results showed that abundant brown PUMA-positive cells were shown in spinal cord cells at day 3 (Figure 4A) and that the number of PUMA-positive cells in spinal cord region of MCC group rats was significantly upregulated (Figure 4B). It is the same region where the compression caused apoptotic cells were observed (Figure 3A). Double staining showed that many but not all PUMA positive cells colocalized with Tuj1 positive neurons and TUNEL labeled cells colocalized well with PUMA (Figure 4C), suggesting that PUMA might be responsible for the apoptosis of both neuronal and non-neuronal spinal cord cells in NIC model.

The mRNA level of PUMA at different days before (day 0) or after surgery (day 1, 3, 7, 14, 28) was examined by qRT-PCR. The most significant up-regulation of PUMA expression was shown at day 3/7 (Figure 4D), and it was the same period we observed the apoptosis in spinal cord cells. Similarly, no significant differences of PUMA expression in CE and DRG were detected before and after compression. Combining the apoptosis and PUMA expression in spinal cord at day 3, we conclude that over-expression of PUMA correlates with compression elicited apoptosis in spinal cord with regional specificity.

### p53 upregulation and SirT2 decrease in compression treated spinal cord

**Figure 5:**
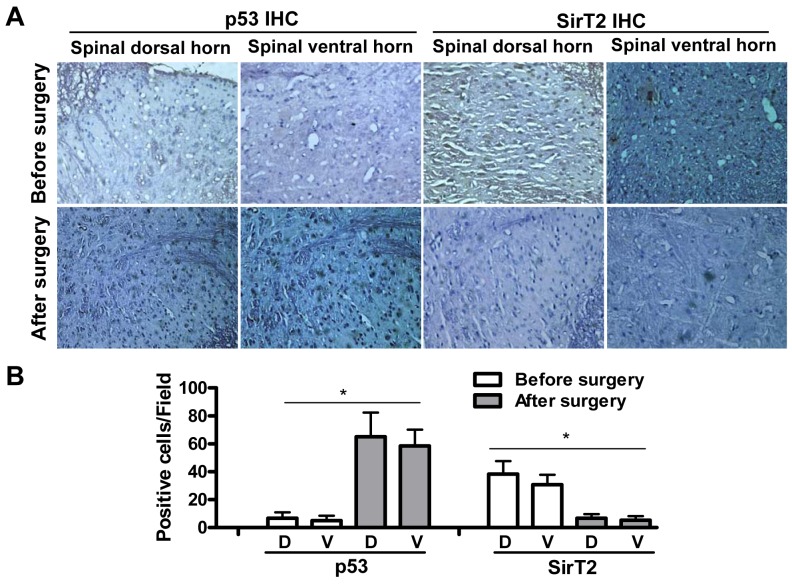
p53 upregulation and SirT2 decrease in compression treated spinal cord. (A) The expression of p53 and SirT2 in spinal dorsal and ventral horn regions was determined by immunohistochemistry (IHC) before (day -3) or after surgery (day 3) in MCC group. (B) At least 5 independent fields of IHC staining were counted and the average numbers of p53- and SirT2-positive cells were shown.

Given that PUMA expression change in spinal cord correlates with compression caused apoptosis, as the important regulators highly related to apoptosis and PUMA, the expression of p53 and SirT2 was determined by immunohistochemical staining in dorsal or ventral horn before and after surgery in MCC animals. It was found that p53-positive cells were rarely observed before surgery, while abundant p53-positive cells were detected in both dorsal and ventral horn of spinal cord at day 3 (Figure 5). Conversely, SirT2-positive cells were observed in spinal cord before surgery and the expression of SirT2 downregulates significantly after surgery in MCC group (Figure 5). It was consistent with the view that SirT2 could negatively regulate p53 activity [11]. Moreover, few visible p53- and SirT2-positive cells were observed in DRG and CE after compression.

### Confirmed expression patterns in spinal cord by Western Blot

**Figure 6:**
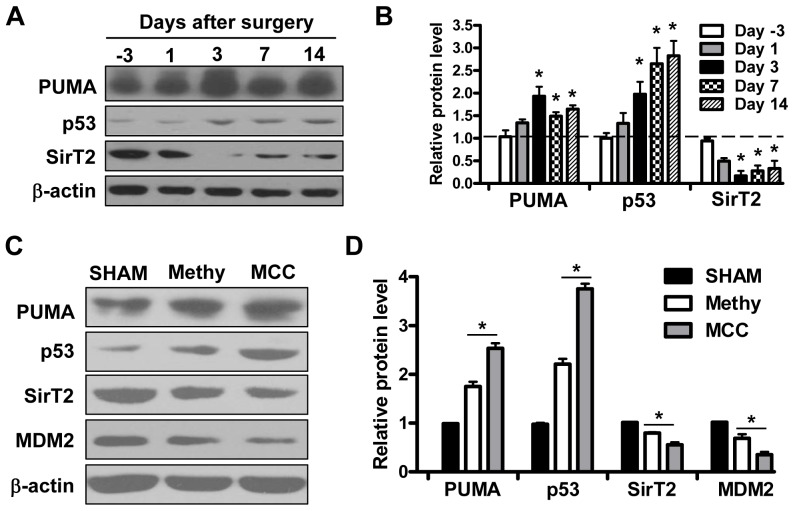
Confirmed expression patterns in spinal cord by Western Blot. (A–B) The expression of PUMA, p53, SirT2 were determined at indicated time points by western blot before or after surgery and β-actin served as loading control. Quantitation of relative band densities was performed and shown in B. (C–D) The expression level of PUMA, p53, SirT2 and MDM2 were evaluated by western blot under Methylprednisolone (Methy) protection in 3 groups: sham-operated group (n = 25); Methylprednisolone (30 mg/kg) combined compression group (n = 36); Modified cauda equina compression group (n = 50). Quantitation of relative protein level was shown in D.

To further confirm these observations, western blot was employed to analyze the protein level of related genes at different time points. It was shown that the expression levels of PUMA and p53 increased after compression especially at day 3 after surgery, simultaneously SirT2 expression downregulates significantly in spinal cord at the same time points (Figure 6A, B). 7 days after surgery, the expression of PUMA, p53 and SirT2 presents the tendency to recover to the level before surgery, which is similar with change tendency of behavioral test (Figure 2).

Furthermore, the expression of PUMA, p53, SirT2 and MDM2 were evaluated in spinal cord when experimental rats were protected by Methylprednisolone (Methy) after compression. We found that compression elicited PUMA and p53 upregulation as well as SirT2 and MDM2 decrease were partially impaired by Methylprednisolone. It further confirmed that compression caused apoptosis in spinal cord of MCC group rats was dependent on p53-PUMA pathway.

## Discussion

In this study, we report a modified model of neurogenic intermittent claudication (NIC) in rats, which was performed by a placement of silicon rubber into L5 and L6 lumbar epidural space. It is simple to be manipulated and reliable to mimic the clinical symptoms in NIC patients. Moreover, the healthy state of rats after surgery keeps well with high survival rate and the present model can reflect the neurophysiological nerve damage, which mimics that of human.

Several rat NIC models have been reported from 1990s. Yamaguchi applied a silicone sheet (3.5×3.5×0.3 mm) to the spinal canal at L4 in a useful rat NIC model [12]. However, the size of silicon sheet is too square for operation. Takenobu implanted two silicon rubber (4.0×1.0×1.25 mm) into the epidural space of L4 and L6 for rat NIC model [4]. The similar result was reported by Sekiguchi et al [3]. Base on these classic NIC models, we modified rat NIC model with proper improvement on rubber size, shape and placement area in rats.

Technically our model requires more simple surgical approach, for it only needs one exposure of L4/6 rather than two exposures of L4/5 and L5/6 in the classic NIC models [4]. It requires shorter operating time for about 10 minutes for MCC model, which leads to less bleeding and faster recovery. We insert silicon rubber under the spinal plate of L5 and L6, which avoids of compression of the conus medullaris. Furthermore, the length of our silicon rubber (10 mm) is standard to maintain the pressure of cauda equina in L5 and L6 and trapezoid-shaped silicon rubber fit the shape of the spinal canal, which is more reasonable to produce suitable degree of compression at L5 and L6 [12] and is much similar to the lumbar spinal stenosis in human [13], [14]. More importantly, current model is able to characterize the development of motor and sensory dysfunction of NIC. As shown in the present study, MCC group rats had lower BBB scores and more sensitive tactile threshold at 3 days after surgery. A significantly shorter running distance was detected in animals after spinal stenosis in free and forced running tests. Based on these advantages, we believe that this modified model could provide more mechanical information in the following study.

Following axon damage peripheral neurons undergo a series of reactive changes including chromatolysis, alterations in protein synthesis and eventually neuronal cell death. Although many studies have focused on the apoptosis of neurons after peripheral nerve injury, little is known about neuronal apoptosis after cauda equina compression and the mechanism of motor dysfunction and sensory disturbance. The neuronal somata in spinal ganglia may be injured and result in degeneration or even death because of Wallerian and retrograde degeneration after nerve root compression [15], [16]. However, the situation of spinal cord after compression is not known.

Apoptosis in dorsal and ventral horn of spinal cord cells was detected in our MCC model, which was consistent with Sekiguchi's report [3]. The observation represents the response of neuronal somata to axon damage, and it suggests that cauda equina compression might cause a series of morphologic, metabolic and expressional changes and injury in spinal cord neurons. The spinal cord cells show characteristic structural abnormities, including folding and capping of the nuclear envelope, displacement of the granular endoplasmic reticulum to the periphery and clustering and enlargement of mitochondria [17], which is the typical characteristics of cell apoptosis.

In this study, the significant increase of PUMA expression was detected 3 days after compression. The results suggested that the response of neuronal somata to axon injury might lead to activation of mitochondria-related apoptosis pathway by abnormal energy metabolism. Consistently, PUMA has been proposed to be a dominant regulator for Bax and Caspase-3 activation in neuronal apoptosis [9], [18]. Here, pro-apoptotic genes Bax, Bak and Bad were upregulated and caspase-3 was activated in MCC group rats under cauda equina compression, which might be caused by PUMA over-expression. It demonstrates that PUMA over-expression is highly associated with apoptosis of spinal cord cells.

Activation of p53 can trigger apoptosis in many cell types including neurons by response to cellular stress as a transcription factor [19]. Furthermore, p53 is also associated with the apoptosis regulated by PUMA [20]. In the modified cauda equina compression model, the expression levels of p53 and PUMA increased significantly in spinal cord cells with region specificity. As p53 inhibitors, MDM2 and SirT2 expression level was decreased simultaneously. These results indicate that PUMA might promote neuronal apoptosis in cauda equina compression through p53-dependent pathway. MDM2 and SirT2 might play positive roles in normal spinal cord tissues through negative regulation of p53 and PUMA expression. Previous report has been suggested that the expression change of specific apoptotic genes possibly serve as a critical change for cells surviving in the chronic pain state [21]. Whereas no direct connection between p53 and pain was established, and this might be our interest in the future study.

Neuronal apoptosis induced by cauda equina compression is the important reason for clinic symptoms of NIC [3]. Understanding of the molecular mechanism will aid in finding the therapeutic target for NIC treatment. The BH-3 domain in PUMA protein plays crucial roles in combination and activation of pro- and anti-apoptotic molecules [9]. Under the stimulation of apoptotic signals, PUMA would translocate from the mitochondria to outer mitochondrial membrane and activate Bax [22], which might subsequently activate caspase-3 in p53-induced apoptotic neurons [23].

In summary, we established a modified rat cauda equina compression model for NIC, which is easy to operate and mimics the typical motor and sensory dysfunction in NIC patients. PUMA was found to be upregulated in compression elicited apoptosis, accompanying the increasing expression of p53 pro-apoptotic Bcl-2 genes as well as the downregulation of SirT2 and MDM2 expression in spinal cord with region specificity. The pro-apoptotic activity of PUMA might be applied as a novel drug target for NIC therapy and PUMA inhibitors could be developed as drug candidates for disease control of nerve injury.
